# Some of the Later Sequelæ of Accidents Occurring during Parturition

**Published:** 1900-07

**Authors:** Edward S. Stevens

**Affiliations:** Lebanon, O.


### SOME OF THE LATER SEQUELÆ OF ACCIDENTS OCCURRING DURING PARTURITION.*

BY EDWARD S. STEVENS, M.D.,

LEBANON, O.

We do and we ought to look upon childbirth as a physiological process. The majority of women pass through it safely, some of them many times, and are all the better for it physically. Many a woman has slight tear of the perineum or of the cervix which, if

* Read before the Miami Valley Medical Society at Loveland, O., May 29, 1900.

left to itself, would heal, leaving the subject of it in as good a condition as before her labor. In these days the *accoucheur* carefully examines his patient for lacerations of the external parts, and when found he generally repairs them at once. Not always, however. The accidents to the parts beyond the reach of the eye are generally not interfered with. Indeed, as often as not the one upon whom the responsibility for the care of these cases rests leaves his patient without so much as knowing that anything untoward has happened. And so it comes to pass that with varying fortunes the young mothers leave their chambers either happy in their new cares with perfect health, or with a greater or less degree of ill health, which they are led to believe is one of the necessary misfortunes of womanhood.

They are not all so. Many a woman leaves her bed apparently well and strong. She is so, indeed, and remains so for years. Then begins a train of symptoms which may begin and end with a profuse and weakening leucorrhea, or there may be uterine and ovarian pains, menorrhagia, peritonitis, sepsis, and even death.

It is to some of these later sequelæ of accidents occurring during parturition to which I will ask your attention for a very few minutes.

The length of time which may elapse after a labor attended by a laceration of not very great extent before symptoms of ill-health directly attributable thereto come on may be almost indefinite, even though after a time cicatrization closes the rent; yet if disease has made a beginning in the torn uterine tissue—disease of such a character that the endometrium becomes involved—granular and bleeding easily, the process will continue after the original cause of disease has disappeared, until it is itself extinguished by cure or self-limitation. These troubles are not often self-limited, however. They proceed to the endometrium of the body of the uterus, to the fallopian tubes, and to the peritoneal cavity beyond. They seal up the fimbriated extremities of the tubes and produce sterility; they cause accumulations in the tubes as tubal abscess, and any of the complications of pelvic inflammation may follow upon these small lacerations at so remote a time as almost to seem to have no connection with them.

To illustrate the minor ailments that may come up as a result of such an accident long after its occurrence, let me speak of the case of Mrs. A. She was the mother of one child four years old when she noticed that she was becoming thin and that she was having stomach trouble. With nothing more than that to go on one would have hesitated about looking to the uterus for the cause. She herself, however, noticed that when her stomach disturbed her most she had a profuse leucorrhea. On examination a bilateral laceration of the cervix was found. This was repaired and most of her unpleasant symptoms left her at once, while her gain in flesh was at the rate of a pound a week for three months. A year and a half later she was delivered of a second child, when the uterus was again torn. After a few months she reported looking thin, having more trouble with her stomach, and having a cough which was not caused by any pulmonary trouble that I could discover. When told that she did not have incipient phthisis, and that a second operation might again relieve her she put it off and has had nothing done, I believe.

Mrs. B.'s case furnishes an example of a little different form of trouble. Her last child was born twelve years ago. Three years ago she began to notice that her menstrual periods were becoming more frequent and that she was having ovarian pains. She entered a hospital in one of the cities near by, where she was advised to have the ovaries removed, but this she declined to do. At the time of my examination I find that she complains of ovarian and tubal tenderness. The cervix is lacerated. She menstruates every two weeks. She declines having anything done that might be called an operation. Hot water twice daily in large amounts relieves the tenderness, but the menstruation continues to be more frequent than natural. Except for this she seems well. I believe the laceration is the cause of her too frequent menstruation and her ovarian distress. A repair of the tear in this case might prevent more serious illness, and perhaps render unnecessary a serious abdominal operation after awhile.

The effect of these disorders upon the menstrual function may be so much more serious than in the case just cited as to become even alarming. Thus in the case of Mrs. C., a badly lacerated cervix was the starting point of disease which did not manifest itself to the patient until her only child was about four years of age. Then her periods became more frequent and more profuse until she had a more or less continuous flow of three months' duration. After proper local and general preparatory treatment the uterus was curetted and the laceration repaired, and the woman restored to comparative health.

In relating this case of Mrs. C. I have not gone into details, and yet her full history would illustrate other features of the subject I am attempting to present which are not without interest. She has been the subject of pelvic inflammation of such a kind and degree that the tubes and ovaries seemed upon examination immovable masses, and the uterus, which was retroverted, was bound firmly in its unnatural position by strong adhesions. She will require further surgical interference to restore these parts to as nearly a natural condition as can be done, but nothing will make them natural. The injury has been done and there is no perfect cure for it.

Another condition to which allusion may be made is sterility. It is not always possible to say when a woman is sterile. I believe, however, that this same Mrs. C. is sterile. A considerable degree of disease may be present, however, without producing sterility, possibly from the fact that but one fallopian tube is infected. In the case of Mrs. D. I believe that this is shown. Her uterus was torn probably in her second confinement. She has had right ovarian pain most of the time since then, more than six years ago, together with leucorrhea, something she never had had before, and considerable weakness. I believe her right fallopian tube was affected about this time, although the supposition is entirely from her history, for she never spoke to me of the matter until within the last few months. Since that time she has given birth to three children, the last one nearly a year ago. During her last pregnancy she suffered greatly from pain in the right side, and since the birth of the last child she not only has suffered from pain in the right side, but the pain had extended to the left side. She has an intermittent discharge of thick yellow "whites"; she complains of irregular chills and slight fever, and in addition to this she suffers from so much weakness that she is almost entirely disabled. It would be interesting to know whether she is now sterile or not, but her condition is so very bad that I would be inclined to believe her to be so. If she lives she will be sterile before so very long, for in my opinion she will not be well until her pelvis is emptied of the diseased masses consequent upon what some persons might call an insignificant tear which nature should be able to care for unaided.

The lessons I would teach by these remarks are to be found in the proper care of the woman during and after confinement. In past times we were told so frequently that "meddlesome midwifery is bad," that the axiom, which was intended to save life and preserve health, has kept many a conscientious physician from doing his whole duty toward his patients. It is true that meddlesomeness is always bad, but carelessness and neglect are just as bad.

Cleanliness is as important in obstetrics as in surgery. The hands of the operator must be clean, and so must the field of operation in either case. The dressings, the clothing, and the bedding must be clean, in both cases. The lying-in room and the nurse must be free from everything that might be a source of infection.

And then as to the accidents proper. I am considering the ordinary tears of the soft parts at this time. Such as may be repaired at once should be. There are small tears of the cervix, however, which cannot be easily recognized at once. The parts should be examined after a period of two or three months, and until this is done the woman should not be considered as dismissed. Where such tears are found after some time has elapsed they are better repaired. No harm may result if they are left, but after considerable length of time many women do suffer because this has not been done.

There is a sequela of these accidents to which I have not alluded, because I believe there is no proof that there is any connection between it and the laceration. I refer to malignant diseases which many students of this branch of medicine consider to be often due to these accidents. It is well enough to bear this thought in mind, however, and to prevent any chance of such disease resulting from one's neglect by repairing or having repaired *all* lacerations of the soft parts.

